# Points in the Ætiology of Puerperal Sepsis

**Published:** 1908-09-19

**Authors:** 


### Obstetrics.

### POINTS IN THE ÆTIOLOGY OF PUERPERAL SEPSIS.

The fact that the mortality from various forms of puerperal sepsis as shown in the Registrar-General's reports does not appear to diminish in a measure proportionate to the advance of aseptic midwifery, is an excuse, if any be needed, for referring to this well-worn subject. The statistics of all well-managed obstetrical clinics show that the incidence of septic diseases, especially of those of the more severe types, can be brought within the narrowest limits; the mortality from puerperal septicæmia in lying-in hospitals is exceedingly small, and then usually occurs in cases which have been treated before being sent into the hospital. Nevertheless, the mortality in the private practices of doctors and midwives still continues. No doubt in the course of a generation or so the effect of the modern training of midwives and students in the principles of aseptic and practical midwifery will be apparent. It is hopeless to look for any marked reduction in the incidence and mortality of puerperal septic diseases until those who still look upon the use of antiseptics as a mere frivolous amusement have retired, and given place to a more enlightened generation.

Even then the study of the ætiological factors involved in the production of puerperal sepsis must be pursued with unabated fervour. For it is only by an intimate knowledge of these factors that certainty in the limitation of septic diseases can be arrived at. We have long been emancipated from the superstition that some mysterious telluric influence is at the bottom of the production of so-called puerperal fever. Puerperal fever, regarded as a nosological entity, is indeed non-existent to-day, and can only be regarded in the same light as other septic manifestations which owe their origin to the infection of wounds by micro-organisms.

Extensive researches have been made from time to time, and still continue to be made, upon the varieties of micro-organisms which may give rise to puerperal infection. As a result of these researches it appears that the organisms most commonly found are the streptococcus pyogenes, the staphylococcus pyogenes aureus and albus, and the bacillus coli communis. These organisms may be present in practically pure culture, or associated one with another, a combined infection by the streptococcus pyogenes and the bacillus coli communis being not at all uncommon. Other organisms, such as the pneumococcus, have been found in the blood in cases of puerperal septicæmia, but according to some recent researches of Bonney and Foulerton the gonococcus is seldom present. In milder cases of septic infection numerous varieties of saprophytic organisms may be found, sometimes in association again with the bacillus coli communis.

This frequent causal connection of the bacillus coli communis with puerperal infection is an outstanding fact of the greatest importance, owing to the ease with which the genital canal can be reached by these organisms. The great practical difficulty in the maintenance of asepsis in midwifery practice is the fact that it is impossible to sterilise the vulva effectively: the skin is too delicate to scrub, and is beset with numberless glands, the ducts and orifices of which harbour organisms. Although in making an ordinary digital vaginal examination it is quite easy to avoid contact with the labia, yet in such manipulations as the introduction of forceps it is impossible to do so. This is the point where the maintenance of rigid asepsis breaks down, owing to the fact that the blade of the forceps, after contact with the labia, probably carries organisms into the higher aseptic regions of the vagina. By care and skill in manipulation such contact can, of course, be reduced to a minimum. Although it is impossible to sterilise the vulva completely, a great deal can be done to render it less septic. The best plan is to wash the parts thoroughly with ether soap and water and then to swab them freely with cyllin or lysol solution. Before this process is undertaken it is wise to empty the rectum by means of an enema, and after the fæces have been evacuated the gut may be washed out with some normal saline solution. This will be effective in preventing the passage of the brownish infected mucus which tends to exude from the rectum during the descent of the head.

Considerable stress should be laid upon the change in the degree of virulence of the infecting organisms in discussions upon the ætiology of puerperal infection. There is no doubt that all pathogenic organisms may pass through periods of greater and of less virulence, and that the severity of the symptoms produced by the organisms is proportionate to their degree of virulence. It is a well-known fact that the virulence of many organisms can be considerably reduced by a series of successive cultivations in artificial media. To raise the organisms to a high degree of virulence it is necessary to pass them through the body of some living animal. In many cases of puerperal infection this principle is unfortunately clearly illustrated. The streptococcus pyogenes is a facultative saprophyte, and can be found in the vagina in large numbers in numerous cases which clinically have all the characteristics of puerperal sapræmia. The streptococci at this period are probably passing through a phase of low virulence, but in virtue of their cultivation in a living body their virulence may increase at any moment, and the organism may develop from a comparatively innocuous saprophyte into an acutely infective pathogenic form. It is really important to bear this fact in mind in dealing with cases of clinical sapræmia, every case of which is potentially a case of septicæmia. In this fact, too, is found one of the clearest indications for dealing with sapræmia without delay and effectively, by cleaning out the uterus and douching its cavity with an antiseptic solution. Many lives are lost through delay in carrying out this necessary procedure, while the medical attendant deludes himself with the text-book idea that the case is only one of sapræmia, and that no grave harm can come of it; from this false sense of security he is wakened rudely and quickly by the transition of the patient into a state of obvious septicæmia.

